# Genomic Fabric Remodeling in Metastatic Clear Cell Renal Cell Carcinoma (ccRCC): A New Paradigm and Proposal for a Personalized Gene Therapy Approach

**DOI:** 10.3390/cancers12123678

**Published:** 2020-12-08

**Authors:** Dumitru A. Iacobas, Victoria E. Mgbemena, Sanda Iacobas, Kareena M. Menezes, Huichen Wang, Premkumar B. Saganti

**Affiliations:** 1 Personalized Genomics Laboratory, CRI Center for Computational Systems Biology, Roy G Perry College of Engineering, Prairie View A&M University, Prairie View, TX 77446, USA; 2 Department of Biology, MD and S Brailsford College of Arts and Sciences, Prairie View A&M University, Prairie View, TX 77446, USA; vemgbemena@pvamu.edu; 3 Department of Pathology, New York Medical College, Valhalla, NY 10595, USA; sandaiacobas@gmail.com; 4 CRI Radiation Institute for Science & Engineering, MD and S Brailsford College of Arts and Sciences, Prairie View A&M University, Prairie View, TX 77446, USA; kmmenezes@pvamu.edu (K.M.M.); huwang@pvamu.edu (H.W.); 5 Department of Physics, MD and S Brailsford College of Arts and Sciences, Prairie View A&M University, Prairie View, TX 77446, USA

**Keywords:** *ALG13*, basal transcription factors, cell cycle, chemokine signaling, *FAM27C*, genomic medicine, kidney cancer, *TASOR*, VEGF signaling, *VHL*

## Abstract

**Simple Summary:**

We applied the genomic fabric principles for personalized gene therapy to a case of clear cell renal cell carcinoma (ccRCC). Despite decades of research, the process of finding the molecular mechanisms responsible for the disease and, more importantly, the therapeutic solution is still a work in progress. We analyzed the transcriptomes of the chest wall metastasis, two distinct cancer nodules, and the cancer-free surrounding tissue in the surgically removed right kidney of a Fuhrman grade 3 metastatic ccRCC patient. The studies revealed that even histopathologically equally classified cancer nodules from the same kidney have different transcriptomic topologies, requiring tailored therapeutic solutions not only for each patient but even for each cancer nodule. We identified death-associated protein kinase 3 (*DAPK3*); transcription activation suppressor (*TASOR*); family with sequence similarity 27, member C, long non-coding RNA (*FAM27C*); and UDP-N-acetylglucosaminyltransferase subunit (*ALG13*) as the gene master regulators of the four profiled regions and proposed molecular mechanisms by which expression manipulation of *TASOR* and *ALG13* may selectively destroy the cancer cells without affecting many of the normal cells.

**Abstract:**

Published transcriptomic data from surgically removed metastatic clear cell renal cell carcinoma samples were analyzed from the genomic fabric paradigm (GFP) perspective to identify the best targets for gene therapy. GFP considers the transcriptome as a multi-dimensional mathematical object constrained by a dynamic set of expression controls and correlations among genes. Every gene in the chest wall metastasis, two distinct cancer nodules, and the surrounding normal tissue of the right kidney was characterized by three independent measures: average expression level, relative expression variation, and expression correlation with each other gene. The analyses determined the cancer-induced regulation, control, and remodeling of the chemokine and vascular endothelial growth factor (VEGF) signaling, apoptosis, basal transcription factors, cell cycle, oxidative phosphorylation, renal cell carcinoma, and RNA polymerase pathways. Interestingly, the three cancer regions exhibited different transcriptomic organization, suggesting that the gene therapy should not be personalized only for every patient but also for each major cancer nodule. The gene hierarchy was established on the basis of gene commanding height, and the gene master regulators *DAPK3,* *TASOR*, *FAM27C* and *ALG13* were identified in each profiled region. We delineated the molecular mechanisms by which *TASOR* overexpression and *ALG13* silencing would selectively affect the cancer cells with little consequences for the normal cells.

## 1. Introduction

The American Cancer Society estimates that by the end of 2020, the USA will have 73,750 (45,520 men, 28,230 women) new cases of kidney (KC) and pelvis cancers, and 14,830 (9860 men and 4970 women) deaths because of these cancers [1]. Smoking (18%), obesity (21%), and hypertension (21%), along with family history are major contributors to the development of the disease, although around 40% of cases cannot be associated with these risk factors [2]. If detected and treated early (while the cancer is confined to the kidney), the survival rate is very high (93% at 5 years). However, for advanced stage, when the cancer has spread to the lungs, brain, bones, and other distant parts of the body, there is only a 12% survival rate at 5 years [1]. The American Joint Committee on Cancer classifies the four KC stages on the basis of the size and extent of the tumor, spread to the nearby nodes, and metastasis to distant cells [3]. In parallel, Fuhrman grade system [4] of KC stages uses the microscopic appearance of the hematoxylin and eosin-stained cells.

Over 90% of KCs arise primarily in the renal parenchyma, with a sTable 2:1 male to female ratio incidence across the population, suggesting possible sexual dimorphism of the genetic susceptibility [5]. The most frequent form of KC is the clear cell renal cell carcinoma (ccRCC) [6]. Despite advancements in detection and treatment, aggressive KC subtypes present challenges due to tumor heterogeneity, biopsy sample availability, different response rates to treatment, and (last but not least) the non-negligible technical noise of the molecular biology assessment methods.

Numerous meta-analyses (e.g., [7,8,9]) have tried to identify the gene biomarkers whose altered sequence and/or expression may serve to diagnose the KC form, and whose restoration may help to cure it. Among others, the 27.0 release (29 October 2020) of the Genomic Data Commons Data Portal [10] includes 59,612 mutations detected in 20,426 genes sequenced from 3295 (2110 male and 1185 female) KC cases. As such, there is practically no gene without mutation reported in at least one case.

Owing to the low frequency in the screened population, no mutated gene emerged as a clear indicator of KC. Thus, only *VHL* (von Hippel–Lindau tumor suppressor, E3 ubiquitin protein ligase; 5.64%)*, PBRM1* (polybromo 1; 4.83%), *TTN* (titin; 4.67%), and *MUC4* (mucin 4, cell surface associated; 2.67%) were mutated in more than 2% of the profiled cases. If instead of all 3295 total KC cases, the mutation frequencies are reported to the 730 cases in the cohort, they increased by a factor of 4.514× (=3295/730): *VHL* (25.48%), *PBRM1*(21.78%), *TTN* (21.51), *MUC4* (12.33%), …, *TP53* (tumor protein p53; 6.58%), …, *MTOR* (mechanistic target of rapamycin; 4.79%), …, *PTEN* (phosphatase and tensin homolog; 4.38%), etc. However, using data from The Cancer Genome Atlas, Rickets et al. [11] concluded that in the cohort of profiled RCC patients, *VHL* was mutated in 41.3% of the cases and *PRBM1* in 38.1%, although neither of these high frequencies was associated with the overall survival. Moreover, a gene can harbor several mutations; for instance, the genes cited above had between 33 (*MTOR*) and 199 (*TTN*) known mutations. Not only are the mutated genes found in almost all chromosomes, but the encoded proteins are involved in a wide diversity of biological processes. Therefore, it is hard to delineate a general molecular mechanism responsible for KC.

*VHL* [12,13], *VEGF* (vascular endothelial growth factor [14]), *CAIX* (carbonic anhydrase IX [15]), *PTEN* [15], *CR1* (complement C3b/C4b receptor 1 [16]), *MERT*K (MER proto-oncogene tyrosine kinase [17]), *PIK3CA* (phosphatidylinositol-4,5-bisphosphate 3-kinase catalytic subunit alpha [18]), and *AKT1* (AKT serine/threonine kinase 1 [19]) are among the most popular KC biomarkers. The PI3K/Akt pathway, one that is altered in several cancers, has been evaluated as a potential target for KC therapy [20,21,22].

However, together with the considered biomarker, hundreds other genes are mutated and/or regulated in KC, and their contributions are neglected without enough evidence that they are verifiably negligible. The “other” affected genes are in unique combinations, not only from patient to patient, but also among cancer nodules in the same tumor (as proven below) and at distant time points during the progression of the disease or recovery of the same person. The collection of the affected genes is unique for each human being because the gathering together of the transcriptomic conditioning factors is never perfectly repeatable. In previous papers, we have shown that the transcriptome depends on genetic background [23], sex [24], age [25], medical history [26], hormonal activity [27], environmental conditions [28], exposure to stress [29], toxins [30], treatment [31], and external stimuli [32]. By evidence, it is impossible to have exactly the same combination of factors and identical influence of a particular factor in distinct individuals. Moreover, even in the same tumor there are histopathologically distinct regions [33,34], a clear indication of different gene expression profiles (distinct cell morphologies result from different expression of cytoskeleton genes and subcellular localization of the encoded proteins). In addition, out of the ≈20,000 genes in the human genome, there are 20 million distinct combinations of two genes and almost 2000 billion of three genes. These numbers make it impossible to assign predictive values to all sets of more than two altered genes. Nevertheless, there are authors who used meta-analysis of large population datasets to predict the diagnostic and therapeutic merits of some particular combinations of two (e.g., [35]) or three (e.g., [36]) gene biomarkers selected for their potential role in the KC development. While the distributions of gene mutations or regulations in large populations of healthy and cancer persons could be statistically distinct, owing to the high inter- and intra-tumor heterogeneity, no gene or combination of few genes can be real predictor(s) for the cancer of a particular individual. In addition, as selected from the most frequently altered genes in large populations, the biomarkers seem to be the most alterable, most likely because they are the genes least protected by the homeostatic mechanisms. Considering that the low players in the cell life are the least protected, restoration of the right sequence/expression level of the biomarkers could be of little consequence, and hence of little therapeutic value.

The genomic fabric paradigm (GFP) [37] offers a holistic alternative to the cancer biomarkers approach by quantifying cancer-induced transcriptome alterations of the genomic fabrics associated with functional pathways. The genomic fabric of a biological process was defined as “the transcriptome associated to the most stably expressed and interconnected gene network responsible for that biological process” [37]. GFP considers the transcriptome as a multi-dimensional mathematical object constrained by a dynamic set of expression correlations among the genes. GFP assigns three independent characteristics to each quantified gene in each phenotype: average expression level, expression variability, and expression coordination with each other gene [38]. GFP paved the way for the gene master regulator (GMR) approach [39] that we believe provides the best targets for cancer gene therapy [40].

## 2. Results

### 2.1. Independent Characteristics of the Quantified Genes in Each Profiled Region

We analyzed previously published gene expression data from samples of chest wall metastasis (CWM), two distinct cancer nodules (denoted by PTA and PTB), and the surrounding normal tissue (NOR) from the right kidney of a ccRCC patient [41]. The 4-biological replicas experimental design provided three independent characteristics for each quantified gene in each region: average expression level (AVE), relative expression variation (REV), and expression coordination (COR) with each other gene (see the Materials and Methods Section 4).

In total, we quantified 13,314 unigenes in each of the 16 profiled samples (4 biological replicates per region). In addition to the 13,314 AVEs that would be considered in any traditional transcriptomic study, we also analyzed 13,314 REVs and (13,314 × 13,313/2 =) 88,624,641 COR values, a tremendous increase (by 6658.5×) of the transcriptomic information for each region.

Expression level, variability, and correlation of genes probed by multiple spots (such as *MIEF1* (mitochondrial elongation factor 1) and *SRRT* (serrate, RNA effector molecule) probed by 20 spots each) were computed as indicated in the Materials and Methods Section 4. Genes not adequately quantified in one sample were eliminated from the analysis.

**Figure 1:**
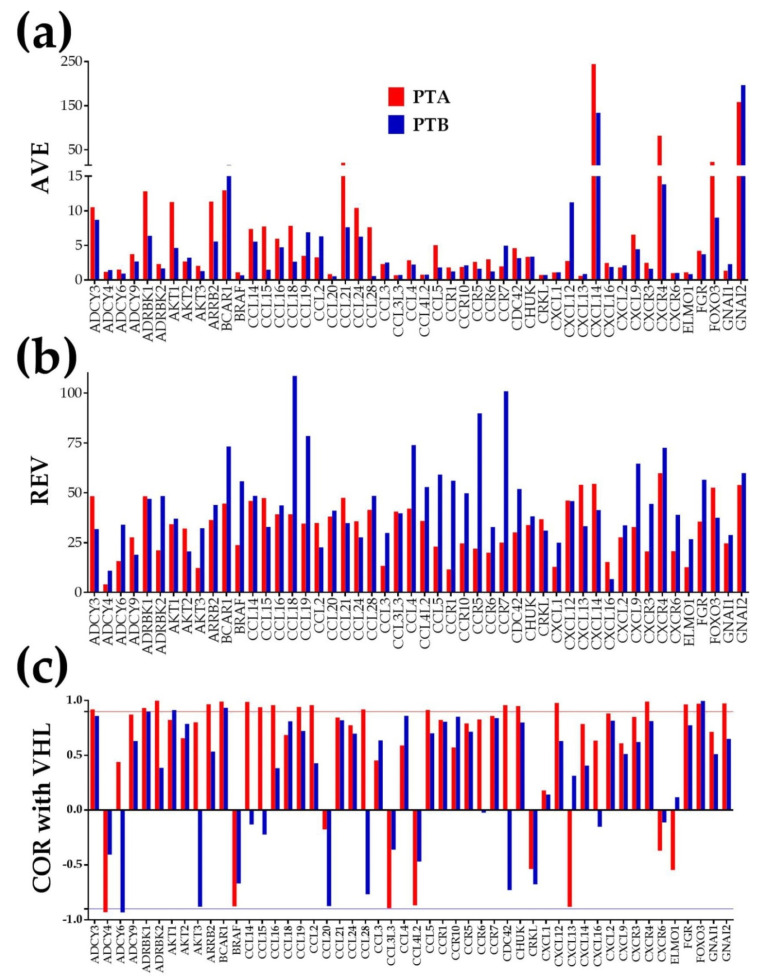
The three independent characteristics of the first 50 alphabetically ordered chemokine signaling genes in the two profiled cancer nodules (PTA and PTB). (**a**) AVE = average expression level; (**b**) REV = relative expression variability; (**c**) COR = correlation coefficient with von Hippel–Lindau tumor suppressor, E3 ubiquitin protein ligase (VHL) Note the independence of the three characteristics in each nodule and the differences between the two nodules despite having the same Fuhrman grade and being isolated from the same (right) kidney. The horizontal lines at COR = −0.95 and COR = 0.95 limit the correlation coefficient interval values out of which the expression coordination is statistically significant (*p* < 0.05).

Figure 1 presents the three independent characteristics in the PTA and PTB regions for the first 50 alphabetically ordered (out of 130) quantified genes involved in the Kyoto Encyclopedia of Genes and Genomes (KEGG)-determined chemokine signaling (CS) pathway [42]. The chemokine signaling pathway was analyzed for the importance of its components in modulating cancer cells’ ability to grow, proliferate, invade, and metastasize (e.g., [43,44]).

*VHL*, a multipurpose adaptor protein, was selected to illustrate the independence of the expression coordination with respect to the average expression level and expression variation because of the alleged role of this gene in KC [12,13,36]. *VHL* is also considered a circuit breaker in the evolution of ccRCC [45]. However, correlation with any other gene should equally justify the independence of the three characteristics.

In addition to the evident independence of the three characteristics of these genes in each region, Figure 1 also shows the differences between the two primary nodules. Thus, only for the illustrated 50 CS genes were *ADCY6*, *AKT1/3*, *CCL15/18/28/5*, *CCR6*, and *CXCL16* found to have significantly higher expression in PTA, and *ADCY4*, *CCL2*, *CXCL12*, and *GNG7* in PTB. There were also substantial differences in REV. Moreover, the number of significantly coordinated chemokines with *VHL* was 19 synergistically and 1 (*ADCY4*) antagonistically expressed in PTA, while in PTB, there were 3 synergistically and 1 (*ADCY6*) antagonistically expressed in PTB. Interestingly, in PTA, *ADCY6* was found to be (not significantly yet) synergistically expressed with *VHL.*

### 2.2. Transcriptomic Differences between Cancer Nodules

**Figure 2:**
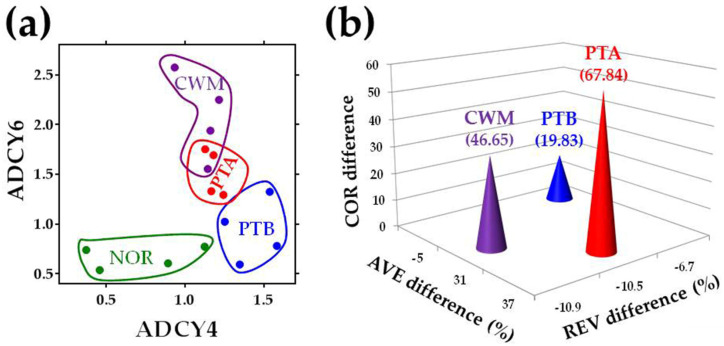
Separation of clear cell renal cell carcinoma (ccRCC) nodules in (**a**) two-gene expression level subspace; (**b**) 3D space of independent measures. (**a**) was limited to the adenylate cyclases 4 (*ADCY4*) and 6 (*ADCY6*), while (**b**) presents the normalized scores for all 130 chemokine signaling genes. Each colored bullet in (**a**) represents the expression levels of the two genes in one of the quarters of the indicated region. The tips of the cones in (**b**) point to the 3D coordinates of the alteration of the quantified genes from the chemokine signaling pathway in the three profiled cancer nodules. Note the clear separation of the three cancer transcriptomes and the shorter distance between PTA and chest wall metastasis (CWM).

Figure 2 illustrates the separation of the NOR, PTA, PTB, and CWM transcriptomes. Figure 2a presents the separation in the two adenylate cyclase genes (*ADCY4*, *ADCY6*) subspace of the expression levels. In this subspace, the center of PTA was at 0.97 expression units from the center of NOR, the center of PTB at 0.77 units, and that of MET at 1.47 units. With respect to NOR, each of the two genes was upregulated in all three cancer regions: *ADCY4* (by 1.66×, 2.01×, 1.57×), *ADC6* (by: 2.29×, 1.40×, 3.14×). However, *ADCY4* had significantly higher expression in PTB than in PTA and CWM, while *ADCY6* had significantly lower expression in PTB.

Figure 2b shows the separation in the 3D space of percentage differences between the cancer nodules and NOR with respect to all three independent variables when all 130 quantified genes from the chemokine signaling pathway and their inter-coordination were considered. Differences with respect to the normal tissue (NOR, placed in the origin of the three orthogonal axes) were computed as indicated in the Materials and Methods Section 4. In this space, the 3D (AVE-diff, REV-diff, COR-diff) coordinates of the cancer nodules are PTA (42.6, −10.5, 103.8), PTB (13.2, −6.7, 18.9), and CWM (59.0, −10.9, 118.8). Thus, PTA, PTB, and CWM were at 112.10, 23.98, and 133.27 units, respectively, from NOR. An interesting result is that while the average expression level and inter-coordination of CS genes increased in all cancer nodules, the expression variability decreased.

Remarkably, although all three cancer nodules were isolated from the same person (PTA and PTB, even from the same kidney) and categorized as the same Fuhrman grade 3 metastatic ccRCC, their transcriptomic alterations with respect to the normal kidney tissue were largely different from each other. Hence, in the 3D subspace limited to only the quantified 130 chemokine signaling genes, the distance between PTA and PTB was 89.9, between PTA and CWM was 22.36, and between PTB and CWM was 110.16. The much smaller distance between CWM and PTA than between CWM and PTB confirms our previous finding from the percentages of the significantly differentially expressed genes [34], showing that CWM cells most likely came from the PTA region.

### 2.3. Regulation of the ccRCC Functional Pathway

**Figure 3:**
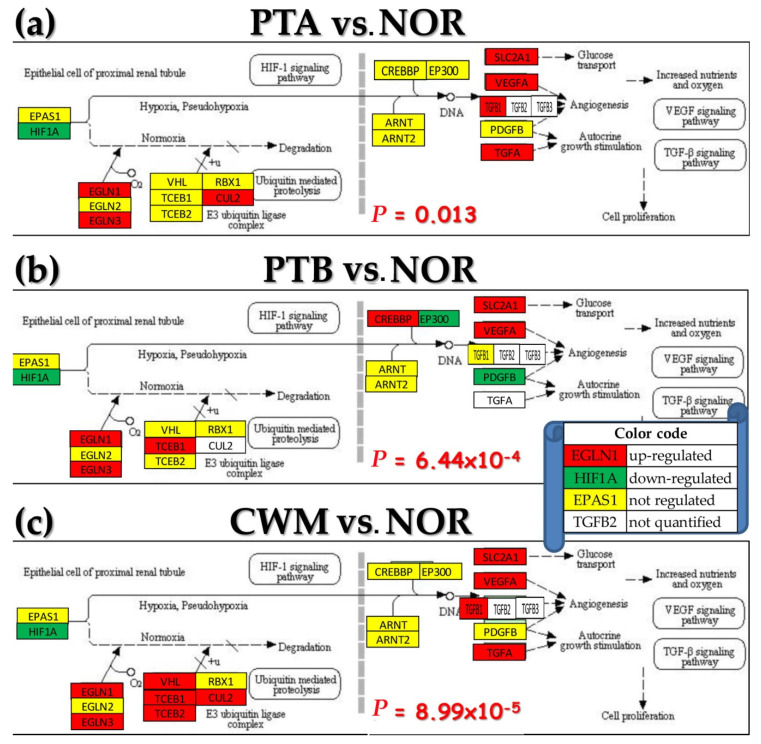
Regulation of the conventional Kyoto Encyclopedia of Genes and (KEGG)-determined ccRCC pathway in the three cancer nodules with respect to the normal tissue from the right kidney. (**a**) PTA vs. NOR; (**b**) PTB vs. NOR; (**c**) CWM vs. NOR Numbers are the *p*-values of the pathway overall regulation in the indicated comparisons. Significantly regulated genes: *CREBBP* (CREB-binding protein), *CUL2* (cullin 2), *EGLN1/3* (egl-9 family hypoxia-inducible factor 1/3), *EP300* (E1A-binding protein p300), *TCEB1/2* (transcription elongation factor B (SIII), polypeptide 1/2), *TGFA/B1* (transforming growth factor alpha/beta 1), *VEGFA* (vascular endothelial growth factor A), and *VHL.*

Figure 3 shows the regulation of the genes responsible for the ccRCC in each of the three cancer nodules with respect to the normal tissue. The genes and their interlinkages were selected from the KEGG-determined human renal cell carcinoma pathway [46]. As indicated by *P* (=*p*-value) computed from hypergeometric distribution of the number of regulated vs. number of quantified genes in the Gene Ontology (GO) term [47], the ccRCC pathway was significantly regulated in all three profiled regions. However, regulation of ccRCC genes was different in the three nodules and even opposite between PTA and PTB for *VEGFA* (vascular endothelial growth factor A).

### 2.4. Regulation of the Apoptosis Pathway

Figure S1 presents the regulation of the KEGG-determined apoptosis pathway [48] in each profiled cancer region with respect to the normal kidney tissue. There were notable differences between the equally ranked cancer nodules PTA and PTB. Thus, there were genes regulated in PTA but not in PTB: *AKT1/3* (v-akt murine thymoma viral oncogene homolog 1/3), *BCL2* (B-cell CLL/lymphoma 2), *IL3RA, IL3RAP* (interleukin receptors), *PRKAR1A* (protein kinases, cAMP-dependent, regulatory, type IA), *RELA* (RELA proto-oncogene, NF-kB subunit), *TNFRSF10A,* and *TNFRSF1A* (members of the tumor necrosis factor receptor superfamily). Other genes were regulated in PTB but not in PTA: *AIFM1* (apoptosis-inducing factor, mitochondrion-associated, 1)*, DFFB* (DNA fragmentation factor B, 40kDa, polypeptide (caspase-activated DNase)), *RIPK1* (receptor-interacting serine/threonine kinase 1), *PIK3R2* (phosphoinositide-3-kinase, regulatory subunit 2 beta), *PPP3CA* (protein phosphatase 3, catalytic subunit, alpha isozyme), *PRKAR1B* (protein kinases, cAMP-dependent, regulatory, type IB), and *TNFRSF10B*. Again, from the perspective of this pathway regulation, CWM was found to be closer to PTA than to PTB.

### 2.5. Regulation of the VEGF Signaling Pathway

**Figure 4:**
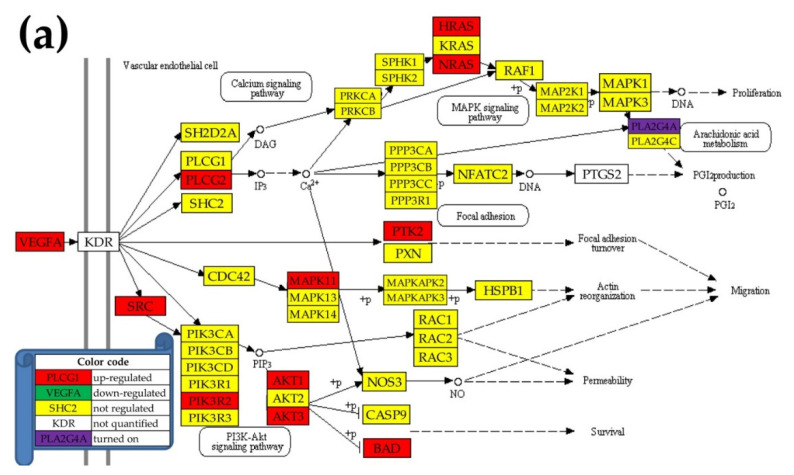

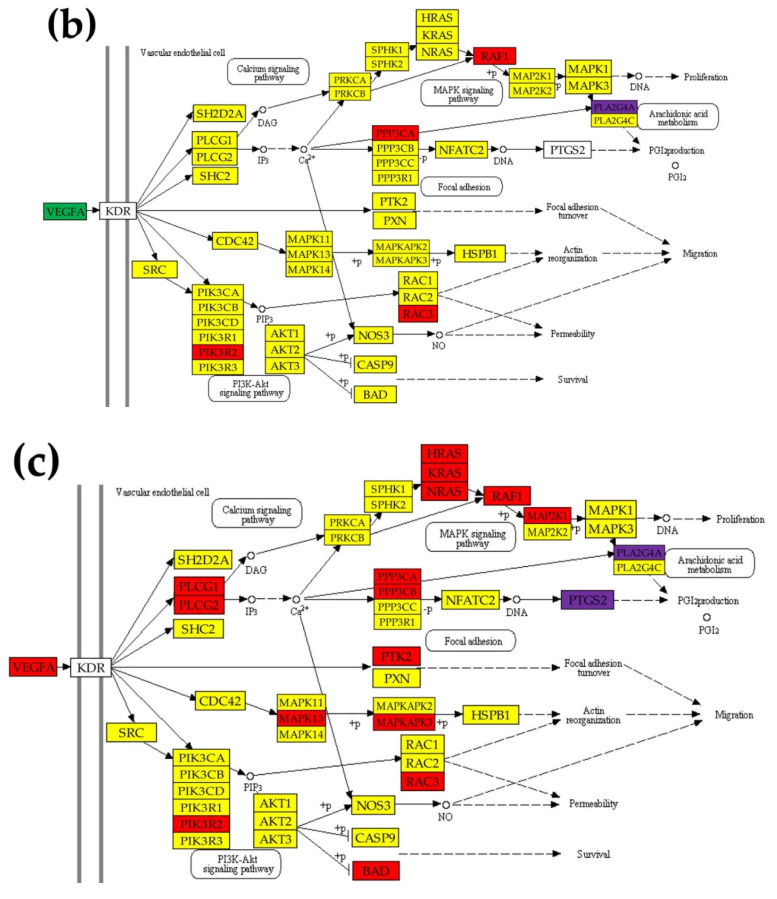
Regulation of the VEGF signaling pathway in (**a**) PTA, (**b**) PTB, and (**c**) CWM. Regulated genes: *HRAS* (Harvey rat sarcoma viral oncogene homolog), *KRAS* (Kirsten rat sarcoma viral oncogene homolog), *MAPK11/13* (mitogen-activated protein kinases 11/13), *NRAS* (neuroblastoma RAS viral (v-ras) oncogene homolog), *PIK3R2* (phosphoinositide-3-kinase, regulatory subunit 2 (beta)), *PLCG2* (phospholipase C, gamma 2 (phosphatidylinositol-specific)), *PTK2* (protein tyrosine kinase 2), and *SRC* (v-src avian sarcoma (Schmidt-Ruppin A-2) viral oncogene homolog).

Figure 4 presents the regulation of the genes involved in the KEGG-determined VEGF signaling pathway [49] in the three cancer regions with respect to the normal kidney tissue. This pathway was analyzed because it is one of the most important targets in KC therapy [50]. Unfortunately, *KDR* (kinase insert domain receptor), the main mediator of the major growth factor (*VEGF*) [51], was not quantified in this experiment. However, we quantified almost all other downstream genes in all regions. Interestingly, in PTA and CWM where *VEGFA* was upregulated, there were substantially more upregulated genes (10 and 15) than in PTB (4), where *VEGFA* was downregulated. While in NOR, *PLA2G4A* (phospholipase A2, group IVA cytosolic, calcium-dependent) was not expressed (AVE(NOR) = 0), in all three cancer regions it was turned on (AVE(PTA) = 0.55, AVE(PTB) = 0.72, AVE(CWM) = 0.54). *PLA2G4A* is considered an important target for prevention and treatment of cancers [52]. Prostaglandin-endoperoxide synthase 2 (prostaglandin G/H synthase and cyclooxygenase) (*PTGS2*), whose upregulation is associated with carcinogenesis and cancer progression [53], was not expressed in any of the three kidney regions (NOR, PTA, PTB) but was turned on in CWM (AVE(CWM) = 0.49).

### 2.6. Alteration of the Homeostatic Mechanisms That Control the Transcript Abundances

The median REVs of the 130 quantified CS unigenes in the four regions were 41.50 (NOR), 37.12 (PTA), 38.73 (PTB), and 36.97 (CWM). Hence, as illustrated in Figure 2b, with respect to NOR, the median REV decreased in PTA by 10.5%, in PTB by 6.7%, and in CWM by 10.9%. Assuming normal distributions of REV values in each region, we found these reductions in the cancer nodules to be highly statistically significant with the respective *p*-values: 3.9 × 10^−17^ (PTA), 5.5 × 10^−4^ (PTB), and 4.5 × 10^−36^ (CWM). REV reduction indicates significant increase of the expression control of CS genes in the cancer cells by the homeostatic mechanisms to confine the fluctuations of the expression levels within narrow intervals, as reported by us in other cancer studies [40,54,55]. The higher median expression variability indicates that PTB region was less altered than the other two cancer regions.

Interestingly, among the CS genes, *PLCB1* (phospholipase C, beta 1) and *RAC1* (ras-related C3 botulinum toxin substrate 1) were three times more variably expressed in PTA than in PTB. However, 14 genes—*CCR1/5/7* (chemokine (C-C motif) receptor 1/5/7)*, GRK6* (G protein-coupled receptor kinase 6), *MAPK3* (mitogen-activated protein kinase 3), *NCF1* (neutrophil cytosolic factor 1)*, PARD3* (par-3 family cell polarity regulator)*, PIK3CG* (phosphatidylinositol-4,5-bisphosphate 3-kinase, catalytic subunit gamma), *PRKCB* (protein kinase C, beta), *ROCK1* (Rho-associated, coiled-coil containing protein kinase 1), *STAT3* (signal transducer and activator of transcription 3 (acute-phase response factor)), *VAV1* (vav 1 guanine nucleotide exchange factor), *WAS* (Wiskott–Aldrich syndrome)*, XCR1* (chemokine (C motif) receptor 1)—were three times more variably expressed in PTB than in PTA. The REV differences indicate again different strengths of the transcripts’ abundance control in the two cancer nodules from the same kidney.

### 2.7. Changes in Gene Networking

**Figure 5:**
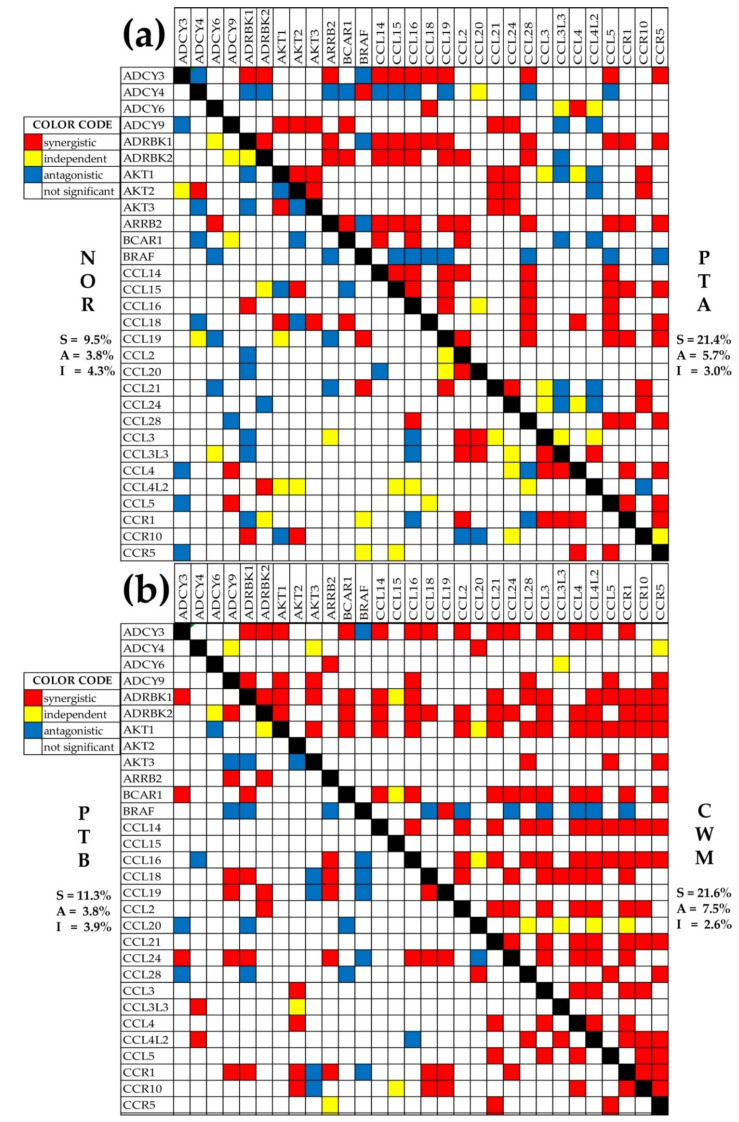
Metastatic ccRCC changed the expression coordination among the chemokine signaling genes. (**a**) Expression correlations in NOR and PTA; (**b**) Expression correlations in PTB and CWM.; The diagonal (black squares) separates the correlation coefficients in the two indicated regions, owing to the symmetry of the Pearson coefficient to the gene permutation. Red/blue/yellow color of a square indicates that the genes labeling the intersecting row and column are significantly synergistically/antagonistically/independently expressed, respectively, while a blank square means that the correlation was not statistically significant. Numbers show the percentage of each type of coordination (S = synergism, A = antagonism, I = independent) for the entire set of 130 chemokine signaling genes.

We found that, with respect to NOR, the number of significantly coordinately (synergistically + antagonistically) expressed chemokine signaling genes with *VHL* increased in PTA with 103.8%, in PTB with 18.9%, and in CWM with 118.8%. These results indicate not only that carcinogenesis reorganizes the gene networking, but also that the network is different from one cancer nodule to another. Remodeling of the transcriptomic networks occurs in all genomic fabrics as well as in all genomic fabrics interplay. Figure 5a,b illustrates the changes in the expression coordination among the first 30 alphabetically ordered chemokine signaling genes. Overall, expression coordination in CWM looked similar to that in PTA, another indication that the CWM cells originated from PTA rather than from PTB. Moreover, although PTA and PTB have the same Fuhrman grade, the expression correlations among the selected genes were largely different, with PTB being closer to NOR.

For some pairs, the significant coordination was switched between synergistic and antagonistic types, as was the case for *ADCY3-CCL5* (negative in NOR, positive in PTA), *ADCY3-CCL28* (positive in PTA, negative in PTB), and *ADCY3-CCL4* (negative in NOR, positive in CWM). These switches indicate significant remodeling of the genomic fabric of the functional pathway, where partner genes that stimulate each other’s expression (positive correlation) in one region become partners that inhibit each other’s expression (negative correlation) in another region. For instance, owing to the positive correlation in PTA, the cancer-associated increase of the chemokine *CCL28* abundance increased the abundance of the linked adenylate cyclase *ADCY3* that regulates numerous pathways, including calcium signaling. By contrast, the negative correlation of the same genes in PTB resulted in a chemokine increase, reducing the abundance of the linked adenylate cyclase. Hence, correlation switches have important downstream consequences.

### 2.8. Changes of the Transcriptomic Landscapes and Genomic Fabrics Interplay

Pair-wise relevance (PWR) analysis [56] was used to determine the transcriptomic landscapes of the KEGG-determined oxidative phosphorylation pathway [57] in all four profiled regions. We found that cancer remodeled the transcriptomic landscapes. Figure S2 illustrates the mitochondrial part of the oxidative phosphorylation landscapes. The analysis showed again that PTA and CWM have similar landscapes, while PTB was found to be closer to NOR. Interestingly, none of the genes from the most relevant five mitochondrial gene pairs in PTA (*ATP6*, *COX2*, *COX3*, *CYTB*), PTB (*COX1*, *COX2*, *COX3*, *CYTB*, *ND2*), and CWM (*COX1*, *COX3*, *CYTB*, *ND4L*) were regulated by cancer in their respective regions. These results indicate that, although the hierarchy and relevance of the gene pairs was affected by the cancer, the expression levels of the most important mitochondrial genes were not necessarily regulated.

### 2.9. Gene Hierarchies and Gene Master Regulators

**Figure 6:**
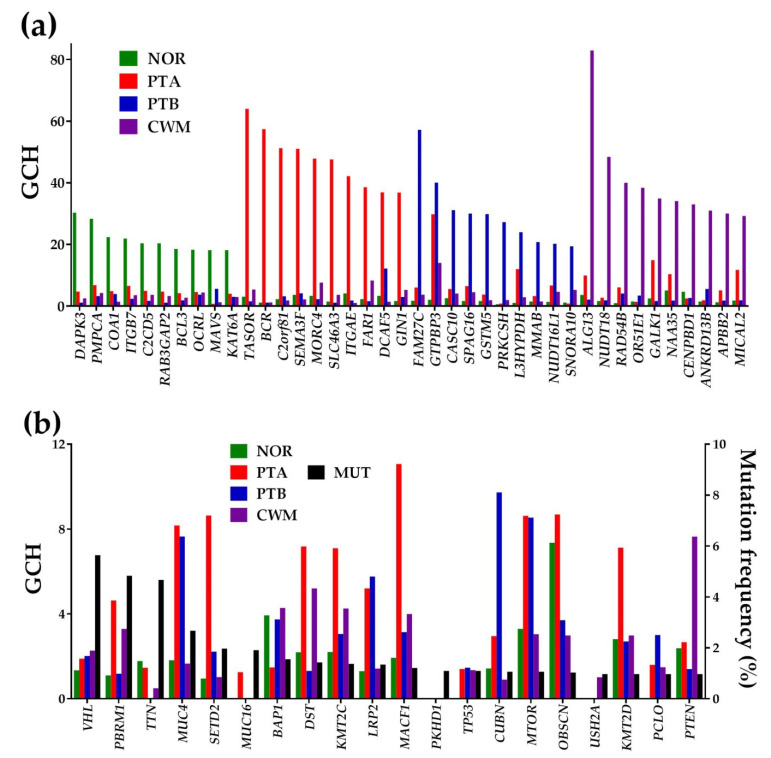
Gene commanding height (GCH) scores of some remarkable genes in each profiled region. (**a**) Top 10 genes in each region and their GCH scores in the other regions. (**b**) GCH scores of the most frequently mutated genes in kidney cancer (mutation frequency on the right axis). Missing columns in (**b**) indicate genes not adequately quantified in that region. GMRs: *DAPK3* (death-associated protein kinase 3) from chromosome 19 in NOR, *TASOR* (transcription activation suppressor) from chromosome 3 in PTA, *FAM27C* (family with sequence similarity 27, member C, long non-coding RNA) from chromosome 9 in PTB, and *ALG13* (UDP-N-acetylglucosaminyltransferase subunit) from chromosome X in CWM. Right axis in (**b**) shows the mutation frequency in the 3295 kidney cancer (KC) cases reported in [10].

We ranked the genes in each profiled region with respect to their gene commanding height (GCH) scores [55,58]. As illustrated in Figure 6, the gene hierarchy was strongly perturbed by the cancer. Note that each region had a different gene hierarchy (no overlap at least for the top 10 genes from Figure 6a). For comparison, Figure 6b presents the GCH scores of the 20 most frequently mutated genes in KC. Remarkably, all of these biomarkers had poor scores in all regions profiled from this patient, far below the GMRs, with the top ranked biomarker, *MACF1* (microtubule-actin crosslinking factor 1), having GCH = 11 in PTA.

It is important to note that the GCH scores of cancer nodules GMRs (transcription activation suppressor (*TASOR*)–64.0; family with sequence similarity 27, member C, long non-coding RNA (*FAM27C*)–57.2; and UDP-N-acetylglucosaminyltransferase subunit (*ALG13*)–83.0) were found to be very low in NOR, indicating that experimental manipulation of these GMRs may have disproportionally larger effects in the cancer regions than in NOR. Thus, targeting the GMR may offer a legitimate solution for the cancer gene therapy [40]. Interestingly, only *FAM27C* was the single upregulated GMR in PTA, with all other three GMRs keeping their normal expression in all regions.

We found also that the GMRs of the four regions are located on different chromosomes (NOR—Chr 19, PTA—Chr 3, PTB—Chr 9, CWM—Chr X), and that they can transcribe in both coding (*DAPK3, TASOR, ALG13*) and non-coding (*FAM27C*) RNAs. Moreover, the GMRs of histopathologically distinct regions may be involved in different pathways. Interestingly, *DAPK3,* the GMR of NOR, is part of the bladder cancer pathway [59], and *ALG13*, the GMR of CWM, is active in the N-glycan biosynthesis [60]. For now, no cancer-related functional pathway has been assigned to *TASOR* and *FAM27C*, with the present study being the first (to our knowledge) to discuss their implications in the development of ccRCC.

### 2.10. GMR at Work

Owing to the death of the patient who provided the profiled samples soon after the surgery (and the lack of U.S. Food and Drug Administration (FDA) approval for the GMR treatment), we were unable to validate the GMRs and determine their therapeutic values. However, we tried to delineate the molecular mechanisms by which the experimental manipulation of a cancer GMR selectively affects the cells it commands with minimal consequences for the normal cells. We used our previously reported finding that significant coordination of one gene with expression of other genes predicts with >80% probability how the coordinately expressed genes will be regulated if the expression of the target gene is significantly altered [54]. This method was validated by stably transfecting two standard human thyroid cancer cell lines (BCPAP and 8505C) with four distinct genes (*DDX19B, NEMP1, PANK2, UBALD1*) and profiling their transcriptome before and after the transfection [39,54].

**Figure 7:**
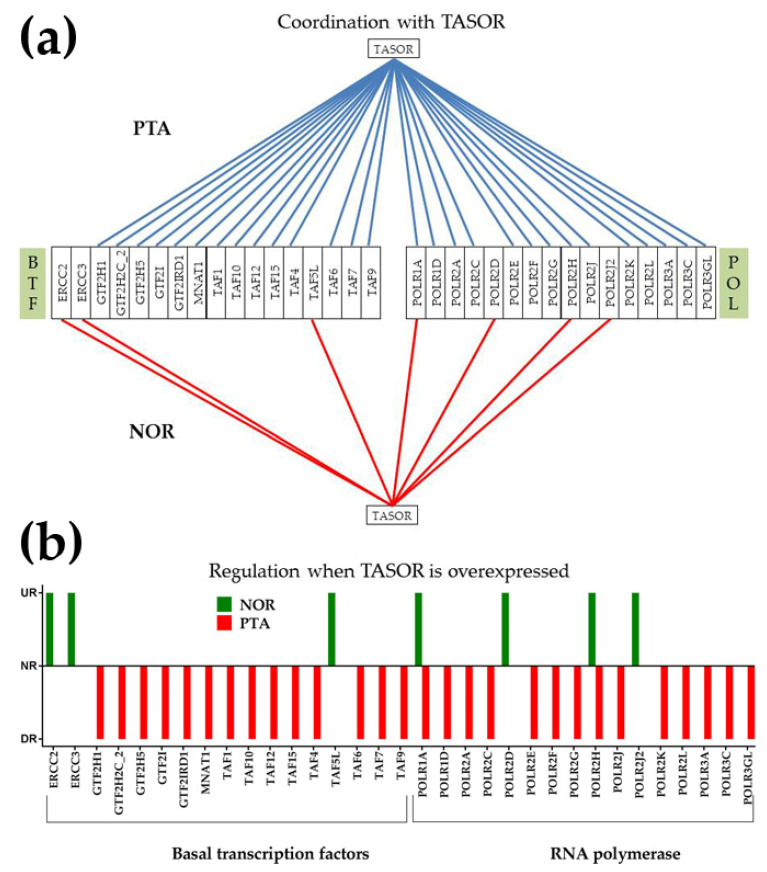
Possible molecular mechanism by which over expression of *TASOR* would selectively destroy the PTA cells. (**a**) Expression correlation of *TASOR* with basal transcription factors (BTF) and RNA polymerase pathway (POL) genes in PTA and NOR. A red/blue line indicates synergistic/antagonistic expression, respectively. Missing line indicates a non-significant correlation. (**b**) Possible effects of *TASOR* overexpression on the basal transcription factors and RNA polymerase pathway genes. UR = upregulated, NR = not regulated, DR = downregulated.

**Figure 8:**
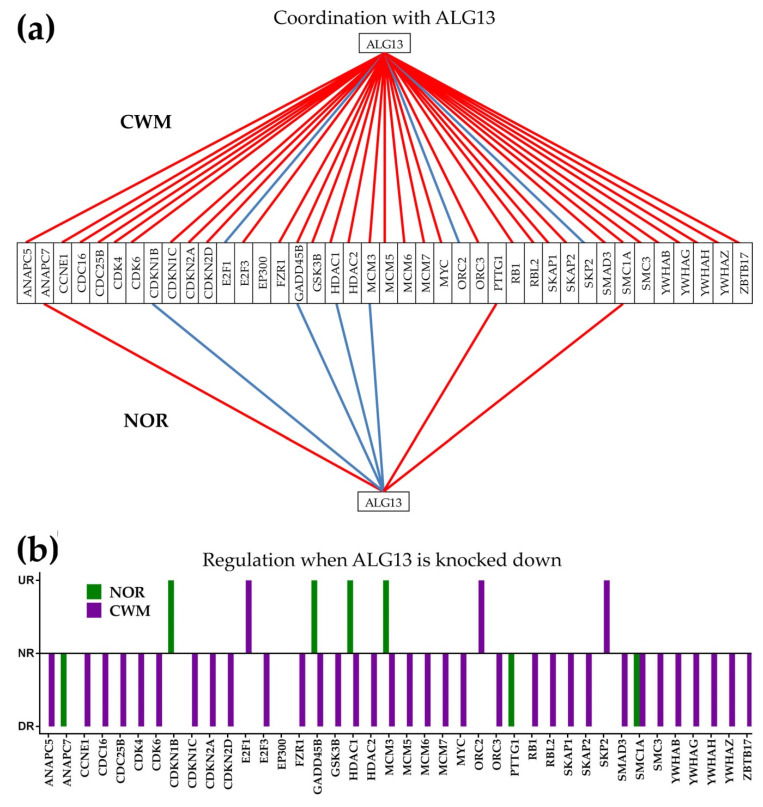
Possible molecular mechanism by which knocking down *ALG13* would selectively destroy the CWM cells. (**a**) Expression correlation of *ALG13* with cell cycle genes in CWM and NOR. A red/blue line indicates synergistic/antagonistic expression, respectively. Missing line indicates non-significant correlation. (**b**) Possible effects of *ALG13* overexpression on the cell cycle genes. UR = upregulated, NR = not regulated, DR = downregulated.

Figure 7a presents possible action mechanisms for *TASOR* in PTA, and Figure 8a presents the possible action mechanisms for *ALG13* in CWM. Figure 7b and Figure 8b show the potential effects of the same GMRs in NOR. We are yet to figure out a likely action mechanism for the non-coding *FAM27C* in PTB. Knowing that cancer cells have a higher survival and growth rate than normal cells, we compared the expression levels and correlations with *TASOR* of the cellular transcriptional machinery in PTA and NOR. Because metastasis is characterized by uncontrollable cell division, we also compared the expression levels and correlations with *ALG13* of the cell cycle (CC) pathway genes in CWM and NOR.

We found that in PTA, 15 out of 36 (42%) quantified basal transcription factors [61] (BTF) and 14 out of 25 (56%) genes from the RNA polymerase pathway (POL) [62] were (*p* > 0.05) significantly antagonistically expressed with *TASOR,* the GMR of that region. No synergistic expression of *TASOR* with genes from these groups was identified (Figure 7a). By contrast, in NOR, 3/36 (8%) BTF and 4/25 (16%) POL genes were synergistically expressed with *TASOR,* but none were antagonistically expressed. Therefore, we hypothesize that a significant overexpression of *TASOR* would downregulate the antagonistically coordinated BTF and POL genes and upregulate its synergistically expressed partners (Figure 7b).

The treatment would correct the expression upregulation of the BTF and POL genes in PTA. There were 12 upregulated BTF genes in PTA: *CDK9*, *ERCC3*, *GTF2F1*, *GTF2H1*, *GTF2H2C_2*, *GTF2H5*, *GTF2IRD1*, *TAF1*, *TAF10*, *TAF3*, *TAF6*, and *TAF6L*, and no downregulated genes. In PTA, there were also eight upregulated POL genes: *POLR1B, POLR1D*, *POLR2A*, *POLR2D*, *POLR2H*, *POLR2J*, *POLR2J2*, and *POLR3GL*, but no downregulated genes. These upregulations indicate that cancer profoundly affects the organization of the cellular transcription machinery and explains the substantial higher growth rate in PTA with respect to NOR.

Interestingly, the statistically significant synergisms of *TASOR* with *POLR1A* and *POLR2H* in NOR were switched to significant antagonisms in PTA. Therefore, overexpression of *TASOR* is expected to have opposite consequences on these two genes in NOR (upregulation) in comparison with PTA (downregulation).

In CWM, the GMR *ALG13* was synergistically expressed with 33 (38%) and antagonistically expressed with 3 (3%) of the 88 quantified KEGG-determined cell cycle (CC) genes [63], while in NOR, 3 (3%) were synergistically and 4 (5%) antagonistically expressed with *ALG13* (Figure 8a). Therefore, we hypothesize that knocking down *ALG13* will significantly slow down the cell cycle (hence cell proliferation) in CWM by downregulating the synergistically expressed CC genes. In NOR cells, acceleration and deceleration caused by knocking down *ALG13* will be practically balanced (Figure 8b), owing to the 3:4 synergistic/antagonistic pairing with *ALG13*.

Again, genes such as *GADD45B* (growth arrest and DNA-damage-inducible, beta)*, HDAC1* (histone deacetylase 1), and *MCM3* (minichromosome maintenance complex component 3) were oppositely coordinated in the cancer nodule in contrast to the normal tissue. Therefore, it is expected that knocking down *ALG13* will downregulate these genes in CWM while upregulating them in NOR. The knocking down of *ALG13* would restore the normal CC pathway in CWM affected by the upregulation of 25% of its genes (0% downregulation). The upregulated CC genes in CWM include the cyclins *CCNB1*, *CCNB1IP1*, *CCNC*, *CCND2*, *CCNY*, *CDK13*, *CDK2AP1*, *CDK4*, *CDK5*, *CDK5R1*, *CDK6*, *CDK9*, *CDKN2A*, *CDKN2D*, *CNNM3*, and *GAK*.

## 3. Discussion

A PubMed [64] search returns thousands (5579 on 11 October 2020) of gene expression studies on ccRCC (among the most recent [65,66,67]), most of them performed on large populations of healthy and cancer subjects. Thus, what novel findings can another study on a single case bring?

Like other groups before, we too have found (but with stronger quantifiers and an absolute fold-change cutoff that takes into account the combined contributions of the biological variability and technical noise) that cancer regulates numerous genes from major functional pathways (apoptosis, basal transcription factors, cell cycle, chemokine signaling, oxidative phosphorylation, renal cell carcinoma, RNA polymerase, VEGF signaling). However, distinct from other reports, our analysis went beyond the traditional expression level by considering the (thousands of times larger) entire information provided by the transcriptomic platform. Thus, we also determined how much the control of the transcript abundance limits the expression variability and the degree by which expression of one gene correlates with expression of each other gene in the same region. In this manner, instead of a compilation of expression levels of N genes, we analyzed a multi-dimensional transcriptome subjected to differential expression control and coordination of individual genes, i.e., N + N + N(N-1)/2 values in each region. In addition to the examples from Figure 1, we proved the independence of the three characteristics in several previous genomic studies on other samples and selections of genes (e.g., [38,54,55]. The analysis of expression variability was possible because the biological replicas collected from the same region can be considered as practically identical systems subjected to slightly different (non-regulatory) local conditions. The correlation analysis is justified by the simultaneous quantification of numerous genes in the same region.

As a good practice in recent genomic studies (e.g., [68]), gene expression profiles of cancer nodules were compared to their correspondent in the surrounding normal tissue. The surrounding cancer-free tissue is the best reference to determine the cancer-related transcriptomic alterations because it is not affected by the biological variability among individual persons.

This study has three major limitations:

(i) We profiled heterogeneous regions composed of several cell types that diluted the transcriptomic alterations assigned to cancer cells. However, profiling separately each cell phenotype is not the solution because the cellular environment is a powerful modulator of the transcriptome. We proved this assertion in previous papers by comparing the gene expression profiles of astrocytes and oligodendrocytes cultured alone and co-cultured in insert systems [58,69]. The cited experiments indicated that the proximity of another (even not touching) cell type significantly regulates the expression and control of numerous genes, and remodels most major functional pathways. In reverse, taking the cell out from its natural environment in the tissue induces non-negligible changes in the cell’s gene expression profile, and thus the tissue transcriptome is not the sum of separately profiled transcriptomes of distinct cell types. Because the therapeutic objective is to cure cancer where it is, the best compromise is to study as homogeneous as possible the small regions, as we did.

(ii) Owing to the death of the patient and the lack of FDA approval, we had no possibility to test the effects of *TASOR* and *ALG13* expression manipulation on cancer nodules and normal tissue. However, we validated that manipulation of the expression of a gene has transcriptomic effects positively correlated with the GCH of that gene [39,40]

(iii) At the time, we also had no possibility to perform additional molecular experiments for functional validation of the investigated pathways. However, most of our transcriptomic results (including tumor heterogeneity [70,71,72,73], activation of chemokine [43,44,74] and VEGF [51,75] signaling pathways, major roles of *TASOR* [76,77] and *ALG13* [78]) can explain functional and clinical observations of other authors.

Nonetheless, one of the most important findings of our study is that each cancer nodule (even from the same kidney) has a unique transcriptomic organization and a distinct gene hierarchy. This finding points out to the necessity to personalize the gene therapy not only for every person [34,73], but even to tailor the therapeutic approach to the characteristics of each major cancer nodule. The conclusion is supported at both individual genes and functional pathways levels by the notable differences in expression level, control, and networking between the equally histopathologically ranked PTA and PTB regions. Importantly, our analyses were able to determine what region of the right kidney metastasized in the chest wall.

The significant increase of the expression levels of chemokines in the cancer nodules is consistent with numerous reports about the chemokine role in cancer progression (e.g., [43,44,74]). The increase of the chemokine signaling was accompanied by increased cell proliferation through upregulation of cyclins and cyclin dependent kinases: 18 in PTA, 11 in PTB, and 17 in MET. *CCNB1IP1, CCNC, CCND2, CDK5R2, CDK9,* and *GAK* were upregulated in all three cancer regions. However, while no cyclin was downregulated in PTA and CWM, five cyclins (*CCNJL, CDK18, CDK19, CDK7, CCNM4*) were downregulated in PTB (up-/downregulated ratio = 11:5). The differences among the three cancer nodules indicate a much faster proliferation of PTA than PTB cells, suggesting that the transcriptomic similarity of CWM and PTA is the result of PTA cells metastasizing in the chest wall. In a previous study [34], we arrived at the same conclusion by determining the percentages of the differentially expressed genes between CWM and PTA (3.6%) and PTB (23.8%) regions.

The difference between the sets of the average expression levels in the two right kidney cancer nodules (PTA and PTB) was not statistically significant (*p* = 0.29), owing to the large dispersion of the results. However, 25 (19%) of the 130 quantified CS genes had significantly different expression levels between PTA and PTB. Moreover, the differences in REVs (*p* = 0.0003) and in correlation coefficients with *VHL* (*p* = 0.0277) were statistically significant. REV dispersion in Figure 1b indicates different strengths of the cellular homeostatic control of transcript abundances among the quantified CS genes in each nodule, as well as differential control of the same chemokine in the two nodules.

Coordination analysis (Figure 1c) revealed again differences among CS genes in the same nodule as well as differences between the two nodules for the same chemokine. The different expression correlations of *VHL* in the two regions point to distinct gene networking [79], raising doubts about the universal structure (independent of race, age, sex, pathological stage, etc.) of the functional pathways determined by KEGG and other specialized software. Altogether, the differences indicate distinct organizational principles [80] of the PTA and PTB transcriptomes. If this is the case for two regions with the same phenotype and isolated from the same kidney, how can one accept a meta-analysis comparing large populations of cancer and healthy patients? As we proved on other tissues, the transcriptome also changes in time because of maturation/ageing [25], development of a disease [28], or in response to a treatment [31]. Therefore, the gene therapy of cancer should be both personalized [81] as well as time-sensitive [40].

A very interesting observation is the highly significant (*p* < 10^−100^) reduction of the expression variability in the cancer nodules with respect to the normal tissue. Thus, when the analysis was applied to all 13,314 quantified unigenes, the median REVs were 41.51 (NOR), 31.45% (PTA), 37.21% (PTB), and 30.46% (CWM). These results confirmed our previous findings in numerous human and animal samples (cited in [58]) that a diseased tissue has a stricter control of the expression level (hence less variability). The robust result of increased expression control is presumably an evolutionary adaptation to limit the disease (here cancer)-related damages.

Figure 2a shows the expression levels of two members of the adenylate cyclase family known to be associated with tumor progression, potential biomarkers for acute myeloid leukemia, and other forms of cancer [82]. Figure 2b shows that the differences between PTA and PTB are not limited to the average expression levels but also to the expression controls and correlations. The significant transcriptomic differences even between equally ranked ccRCC nodules in the same kidney (illustrated in Figure 2) confirm the intratumoral heterogeneity [70,71,72,73]. The differences also raise serious doubts about coding [81] or non-coding [83] the transcriptomic signature of a particular form of renal cancer in all persons [84,85].

The transcriptomic differences among the profiled regions can be partially caused by the differential RNA editing activity [86]. We believe that RNA editing is sensitive to many local factors whose combination cannot be exactly repeated even in distinct regions of the same tumor. The differences between PTA and PTB were also evident in Figure 5, showing the inter-coordination of chemokine signaling genes. We also found distinct transcriptomic PWR landscapes in the two regions (Figure S2b,c) and different gene hierarchies (Figure 6a), with no overlap among the top 10 genes. However, the common Fuhrman grade assigned to the three cancer nodules by the pathology report of the profiled samples indicates transcriptomic redundancy of the histological features (i.e., histopathology compatible with several transcriptomic organizations).

Correlation analysis relies on the “principle of transcriptomic stoichiometry” that requires genes whose encoded products are related in a functional pathway be coordinately expressed to optimize the pathway [38,54]. We used the coordination analysis to identify the molecular mechanisms by which manipulation of cancer GMRs may selectively affect the cancer cells. *TASOR* (also known as *FAM208A*), the GMR of PTA, reported to be an important player for cell division [77], was synergistically correlated with *POLR1A* and *POLR2H* in NOR but antagonistically expressed with the same genes in PTA. *ALG13*, an important modifier of renal filtration defects [78], was oppositely coordinated with *GADD45B*, *HDAC1*, and *MCM3* in CWM to that of NOR. These opposite correlations indicate the strong dependence of the gene interactions on the cellular phenotype [58] and genetic background [23] that are disregarded by the functional pathways built by popular software (KEGG, Ingenuity, DAVID, GennMapp, etc.).

On the basis of the correlation analysis, we speculate (without functional validation) that overexpression of *TASOR* would have the following major effects in PTA:

—Downregulation of three members of the general transcription factor IIH, polypeptide (*GTF2H1/2C_2/5).* Degradation of *GTF2H1* (a.k.a. *P62*) was associated with enhanced apoptosis and autophagy [87].

—Downregulation of the TATA-box-binding protein associated factors *TAF1*, *TAF10*, *TAF12*, *TAF6*, *TAF7*, and *TAF9*, all essential for the initiation of transcription by RNA polymerase II [88] and to constructing multi-protein complexes.

—Downregulation of two components of RNA polymerase I complex (*POLR1A*, *POLR1D*) that transcribe DNA into ribosomal RNA (rRNA) precursors. The rRNA precursors are fundamental for ribosome biogenesis and protein synthesis, and their inhibition limits cellular growth and proliferation [89].

—Downregulation of nine components (*POL2RA*, *POL2RC*, *POL2RE*, *POL2RF*, *POL2RG*, *POL2RH*, *POL2RJ*, *POL2RK*, *POL2RL*) of the RNA polymerase II, responsible for synthesizing mRNA. It was recently reported that *POL2RA* silencing via siRNA (small interference RNA) is a treatment for triple-negative breast cancer and induces a substantial reduction of tumor growth [90]. Our study shows that *POL2RA* could be also a target for ccRCC gene therapy.

—Downregulation of three (*POLR3A*, *POLR3C*, *POLR3GL*) catalytic components of RNA polymerase III, which synthesize small RNAs and were suggested as potential targets for breast cancer [91].

By contrast, in NOR, overexpression of *TASOR* is expected to:

—Upregulate *ERCC2/3* (excision repair cross-complementation group 2/3);

—Upregulate TAF5L POLR1A, POLR2D, POLR2H, and POLR2J.

In summary, overexpression of *TASOR* would substantially reduce the transcription in cancer cells (hence their proliferation) while increasing the renewal of the normal cells.

Correlation analysis further indicated that silencing *ALG13* via siRNA or CRISPR (clustered regularly interspaced short palindromic repeats) would downregulate numerous cell cycle genes in CWM while keeping a relative balance in NOR. High expression of *ALG13* was associated with poor overall survival in non-small cell lung cancer [92]. Thus, silencing *ALG13* would have both direct and indirect (mediated by CC genes) on the cancer cells. The subset of CC genes expected to be downregulated in CWM includes:

—*ANAPC5* (anaphase-promoting complex subunit 5), which regulates cell cycle progression by ubiquitinating cell cycle proteins for proteolysis by the proteasome [93];

—One cyclin (*CCNE1*), two cell division cycles (*CDC16*, *CDC25B*)*,* two cycle-dependent kinases (*CDK4, CDK6*), three cycle-dependent kinase inhibitors (*CDKN1C*, *CDKN2A*, *CDKN2D*);

—Four components of the minichromosome maintenance complex (*MCM3*, *MCM5*, *MCM6, MCM7*) and four tyrosine 3-monoxygenase/tryptophan 5-monoactivation proteins (*YWHAB*, *YWHAG*, *YWHAH*, *YWHAZ*).

All these synergistically expressed genes with *ALG13* in CWM are critical for the cell cycle progression and their downregulation may induce unbearable alterations of the cell transcriptome.

We recommend that clinical implementation of the GMR personalized cancer gene therapy follows the following steps:

(1) Take a small biopsy from the cancer nodule of a solid tumor or collect a circulating tumor;

(2) Split the biopsy into four parts (at least four biological replicas are needed for the accurate determination of the expression correlations);

(3) Profile separately the transcriptome of each quarter with a reliable, low-noise, high-throughput gene expression platform (microarray or RNA-sequencing);

(4) Use the Cancer—GMR software [40] to establish the gene hierarchy and identify the GMR;

(5) If the GMR does not have significantly larger GCH than the next gene(s), consider the first 2–3 genes to manipulate;

(6) Select the method to manipulate (overexpressing or silencing) the expression of the GMR(s) pending on its molecular function(s) and correlations with major functional pathways;

(7) Build/buy the construct to change the expression of the GMR(s);

(8) Introduce the gene construct in the tumor or in the systemic circulation.

Because the gene hierarchy is unique for each patient, there is no universal solution for everybody. However, there are already commercially available constructs for overexpression and/or silencing of several genes. In time, the industry will produce shelf-ready constructs for almost all genes, which will considerably reduce not only the costs of the therapy but also the interval from genomic diagnostic to the treatment application.

## 4. Materials and Methods

### 4.1. Gene Expression Data

Publicly available raw and processed gene expression data from a 74-year-old male who had undergone total right kidney nephrectomy and resection of a chest wall soft tissue mass were downloaded from [41]. CWM was isolated from the center of the chest wall tumor. PTA and PTB were isolated from two distal regions of the 5.5 primary tumor, and NOR from the cancer-free resection margins of the right kidney. All three cancer nodules were assigned Fuhrman grade 3 in the pathology report. The transcriptomic profiles were obtained by using Agilent 4 × 44 K two-color microarray (platform GPL13497 [94]. From each region, we collected under the microscope a ≈2 mm^3^ sample (as histologically homogeneous as possible), which was further split into 4 parts, with each quarter being profiled separately as a biological replica. The results from NOR served as the reference for changes in the 3 cancer regions. Corrupted spots or with foreground fluorescence less than twice the background in at least one sample were discarded from analysis.

### 4.2. Average Expression Level (AVE)

For each gene in each region, we computed the average expression level of the 4 replicates considering the redundancy of the microarray spots probing the same gene:

(1) $$\begin{array}{l} AVE_{i}^{\left(region\right)}=\frac{1}{R_{i}}\sum_{k=1}^{R_{i}}\mu_{i,k}^{\left(region\right)}=\frac{1}{R_{i}}\sum_{k=1}^{R_{i}}\left(\frac{1}{4}\sum_{j=1}^{4}a_{i,k,j}^{\left(region\right)}\right)\;,\;where:\\ region=NOR,PTA,PTB,CWM\\ R_{i}=\;\mathrm{number}\;\mathrm{of}\;\mathrm{spots}\;\mathrm{probing}\;\mathrm{redundantly}\;\mathrm{gene}\;i\\ a_{i,k,j}^{\left(region\right)}=\mathrm{expression}\;\mathrm{level}\;\mathrm{of}\;\mathrm{gene}\;“i”\;\mathrm{probed}\;\mathrm{by}\;\mathrm{spot}\;“k”\;\mathrm{on}\;\mathrm{biological}\;\mathrm{replica}\;“j”\;\mathrm{in}\;“\mathrm{region}”. \end{array}$$

### 4.3. Relative Expression Variability

Owing to the non-uniform redundancy of probing spots in the microarray, we replaced the coefficient of variation “CV” with the Bonferonni-like corrected mid-interval of the chi-squared estimate of the pooled CV for all quantifiable spots probing redundantly the same transcript:

(2) $$\begin{array}{l} REV_{i}^{\left(region\right)}=\underset{\mathrm{correction}\;\mathrm{coefficient}}{\underset{︸}{\frac{1}{2}\left(\sqrt{\frac{r_{i}}{\chi^{2}\left(r_{i};0.975\right)}}+\sqrt{\frac{r_{i}}{\chi^{2}\left(r_{i};0.025\right)}}\right)}}\sqrt{\underset{\mathrm{pooled}\;\mathrm{CV}}{\underset{︸}{\frac{1}{R_{i}}\sum_{k=1}^{R_{i}}{\left(\frac{s_{ik}^{\left(region\right)}}{\mu_{ik}^{\left(region\right)}}\right)}^{2}}}}\times 100\%\\ \mu_{ik}\;=\;\mathrm{average}\;\mathrm{expression}\;\mathrm{level}\;\mathrm{of}\;\mathrm{gene}\;i\;\mathrm{probed}\;\mathrm{by}\;\mathrm{spot}\;k\;(=\;1,\;\ldots ,\;R_{i})\;\mathrm{in}\;\mathrm{the}\;4\;\mathrm{biological}\;\mathrm{replicas}\\ s_{ik}=\mathrm{standard}\;\mathrm{deviation}\;\mathrm{of}\;\mathrm{the}\;\mathrm{expression}\;\mathrm{level}\;\mathrm{of}\;\mathrm{gene}\;i\;\mathrm{probed}\;\mathrm{by}\;\mathrm{spot}\;k\\ r_{i}=4R_{i}-1=\;\mathrm{number}\;\mathrm{of}\;\mathrm{degrees}\;\mathrm{of}\;\mathrm{freedom}\\ R_{i}\;=\;\mathrm{number}\;\mathrm{of}\;\mathrm{microarray}\;\mathrm{spots}\;\mathrm{probing}\;\mathrm{redundantly}\;\mathrm{gene}\;i \end{array}$$

### 4.4. Expression Coordination

The expression coordination of 2 genes in the same region was evaluated with the Pearson pair-wise correlation coefficient *ρ_ij_* between their (log_2_) expression levels in the 4 biological replicas. The statistical significance of the correlation was determined with the two-tail *t*-test for the 4 (biological replicas) × R (number of spots probing the same gene)—2 degrees of freedom [95].

### 4.5. Expression Regulation

A gene was considered as significantly regulated in a cancer nodule (PTA, PTB, CWM) with respect to NOR, or differentially expressed between the two primary tumors PTA and PTB if

(3) |xi(A→B)|>CUTi=1+11002((REVi(A))2+(REVi(B))2) ^ pval<0.05where:A=NOR,PTA , B=PTA,PTB,CWMxi(A→B)≡{μi(B)μi(A), if μi(B)>μi(A)−μi(A)μi(B), if μi(B)<μi(A) , μi(A/B)=1Ri∑k=1Riμik(A/B)

### 4.6. Transcriptomic Separation

The transcriptomic distance (TD) for a given pathway Γ between 2 profiled regions (region 1, region 2) was determined using Pythagoras’ theorem in the Euclidian space of the 3 orthogonal (independent) variables:

(4) $$\begin{array}{l} TD_{\Gamma }^{\left(region2-region1\right)}=\sqrt{\left({\left(AVEdiff\right)}^{2}+{\left(REVdiff\right)}^{2}+{\left(CORdiff\right)}^{2}\right)}\times 100\%\\ \mathrm{where}:\;\{\Gamma \}\;\mathrm{is}\;\mathrm{the}\;\mathrm{number}\;\mathrm{of}\;\mathrm{genes}\;\mathrm{in}\;\mathrm{the}\;\mathrm{pathway}\;\Gamma \\ AVEdiff\equiv \frac{1}{\left\{\Gamma \right\}}\sum_{i=1}^{\left\{\Gamma \right\}}\left(\frac{AVE_{i}^{\left(region2\right)}}{AVE_{i}^{\left(region1\right)}}-1\right)\\ REVdiff\equiv \frac{1}{\left\{\Gamma \right\}}\sum_{i=1}^{\left\{\Gamma \right\}}\left(\frac{REV_{i}^{\left(region2\right)}}{REV_{i}^{\left(region1\right)}}-1\right)\\ CORdiff\equiv \frac{1}{\left\{\Gamma \right\}}\sum_{i=1}^{\left\{\Gamma \right\}}\left(\frac{COR_{i,VHL}^{\left(region2\right)}}{COR_{i,VHL}^{\left(region1\right)}}-1\right) \end{array}$$

### 4.7. Pair-Wise Relevance

The pair-wise relevance (PWR, [38]) of genes *i* and *j* in the region *R* = NOR, PTA, PTB, CWM was computed as

(5) $$\begin{array}{l} PWR_{ij}^{\left(region\right)}=\frac{AVE_{i}^{\left(region\right)}AVE_{j}^{\left(region\right)}}{{\left(\bar{AVE^{\left(region\right)}}\right)}^{2}}\times {\left(\rho_{ij}^{\left(region\right)}\right)}^{2}\times \frac{{\left(\bar{REV^{\left(region\right)}}\right)}^{2}}{REV_{i}^{\left(region\right)}REV_{j}^{\left(region\right)}}\;,\;where:\\ region=NOR,PTA,PTB,CWM\\ \bar{AVE^{\left(region\right)}}\;=\;\frac{1}{N}\sum_{k=1}^{N}AVE_{k}^{\left(R\right)}\;,\;N=\mathrm{number}\;\mathrm{of}\;\mathrm{unigenes}\;,\;\bar{REV^{\left(region\right)}}\;=\;\frac{1}{N}\sum_{k=1}^{N}REV_{k}^{\left(region\right)}\\ \rho_{ij}^{\left(region\right)}=\;\mathrm{Pearson}\;\mathrm{correlation}\;\mathrm{beween}\;\mathrm{the}\;\mathrm{expression}\;\mathrm{levels}\;\mathrm{of}\;\mathrm{genes}\;i\;\mathrm{and}\;j\;\mathrm{in}\;\mathrm{region}\;“\mathrm{region}”. \end{array}$$

### 4.8. Gene Commanding Height (GCH) and Identification of Gene Master Regulator (GMR)

We established the gene hierarchy in each region on the basis of their gene commanding height (GCH) score [39,54]:

(6) $$\begin{array}{l} GCH_{i}^{\left(region\right)}=\underset{\mathrm{transcription}\;\mathrm{control}\;\mathrm{estimate}}{\underset{︸}{\frac{{\langle REV\rangle }^{\left(region\right)}}{REV_{i}^{\left(region\right)}}}}\times \underset{\mathrm{measure}\;\mathrm{of}\;\mathrm{expression}\;\mathrm{coordination}}{\underset{︸}{\exp\left(4{\bar{{\left(\rho_{ij}^{\left(region\right)}\right)}^{2}}|}_{\forall j\neq i}\right)}},\;where:\\ \langle \;\rangle \;=\;\mathrm{median},\;\bar{{\left(\;\right)}^{2}}\;=\;\mathrm{average}\;\mathrm{of}\;\mathrm{the}\;\mathrm{square}\;\mathrm{values}. \end{array}$$

The gene master regulator (GMR, [40]) of the region “*region*” is the top gene (highest GCH) of that region.

## 5. Conclusions

This study presents the principles of a new kind of systemic therapy of renal carcinoma [96]. The proposed GMR approach identifies in each cancer nodule of each patient the gene master regulators (GMR), whose “smart” expression manipulation would selectively destroy the cancer cells (and only them) wherever they are in the body. The personalized and time-sensitive GMR therapy differs essentially from the popular cancer biomarkers approach that looks for time-independent, universal (good for everybody) treatment (e.g., [97,98]).

## Supplementary Materials

The following are available online at https://www.mdpi.com/2072-6694/12/12/3678/s1. Figure S1: Regulation of the apoptosis pathway in (a) PTA, (b) PTB, and (c) CWM regions with respect to NOR, Figure S2: The PWR landscapes of mitochondrial genes in the four profiled regions.
