# Supplementary material for: Genomic Fabric Remodeling in Metastatic Clear Cell Renal Cell Carcinoma (ccRCC): A New Paradigm and Proposal for a Personalized Gene Therapy Approach
Source: Cancers (Basel). 2020 Dec 8;12(12):3678. doi: 10.3390/cancers12123678 (PMC7762545; doi:10.3390/cancers12123678)
Supplement: Supplementary file 1 [file cancers-12-03678-s001.pdf]

Supplementary Material

Genomic Fabric Remodeling in Metastatic Clear Cell Renal Cell Carcinoma (ccRCC): A New Paradigm and Proposal for a Personalized Gene Therapy Approach

Dumitru A. Iacobas, Victoria E. Mgbemena, Sanda Iacobas, Kareena M. Menezes, Huichen Wang and Premkumar B. Saganti

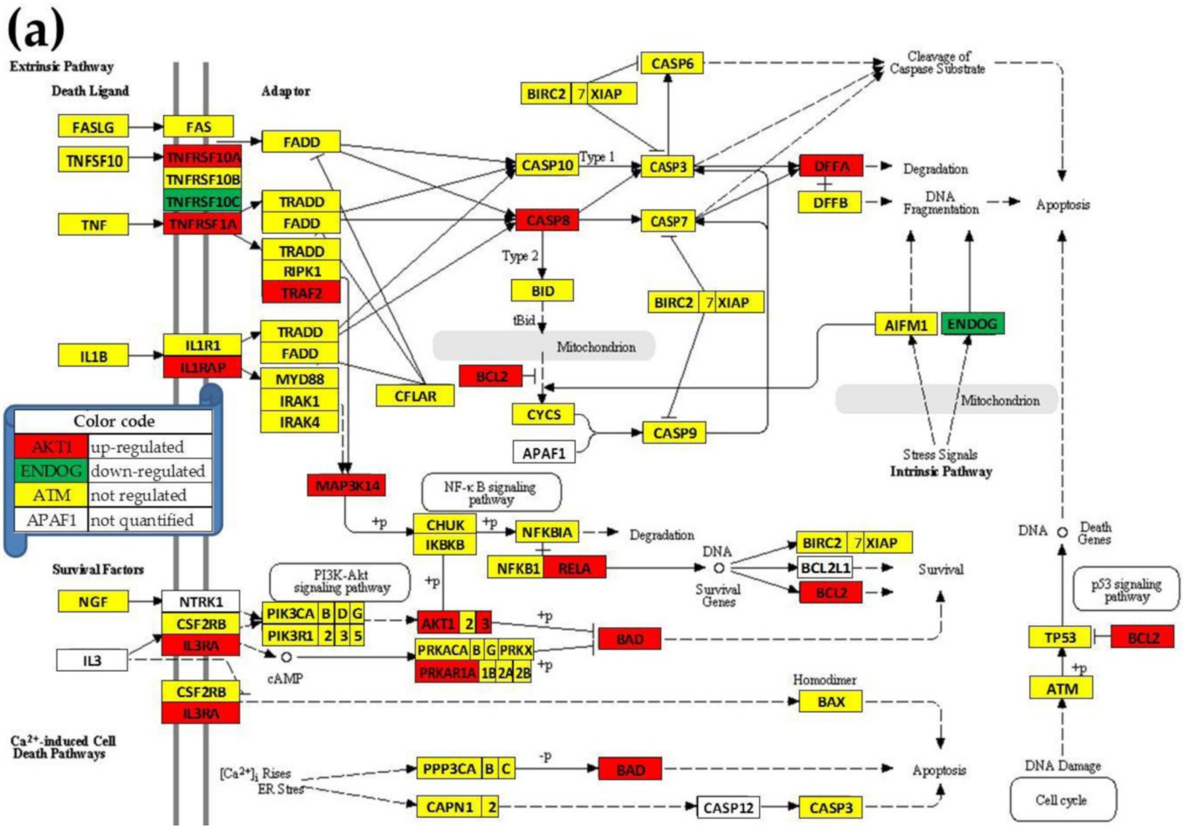

(b)

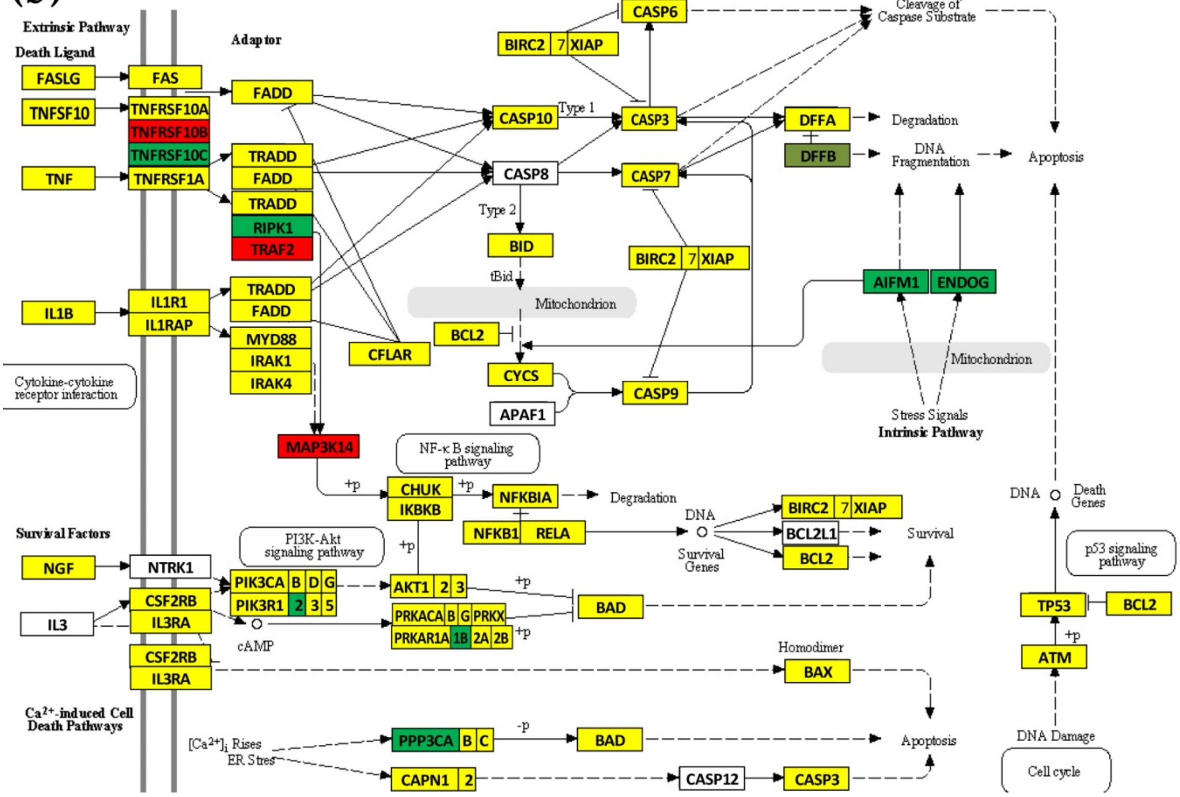

(c)

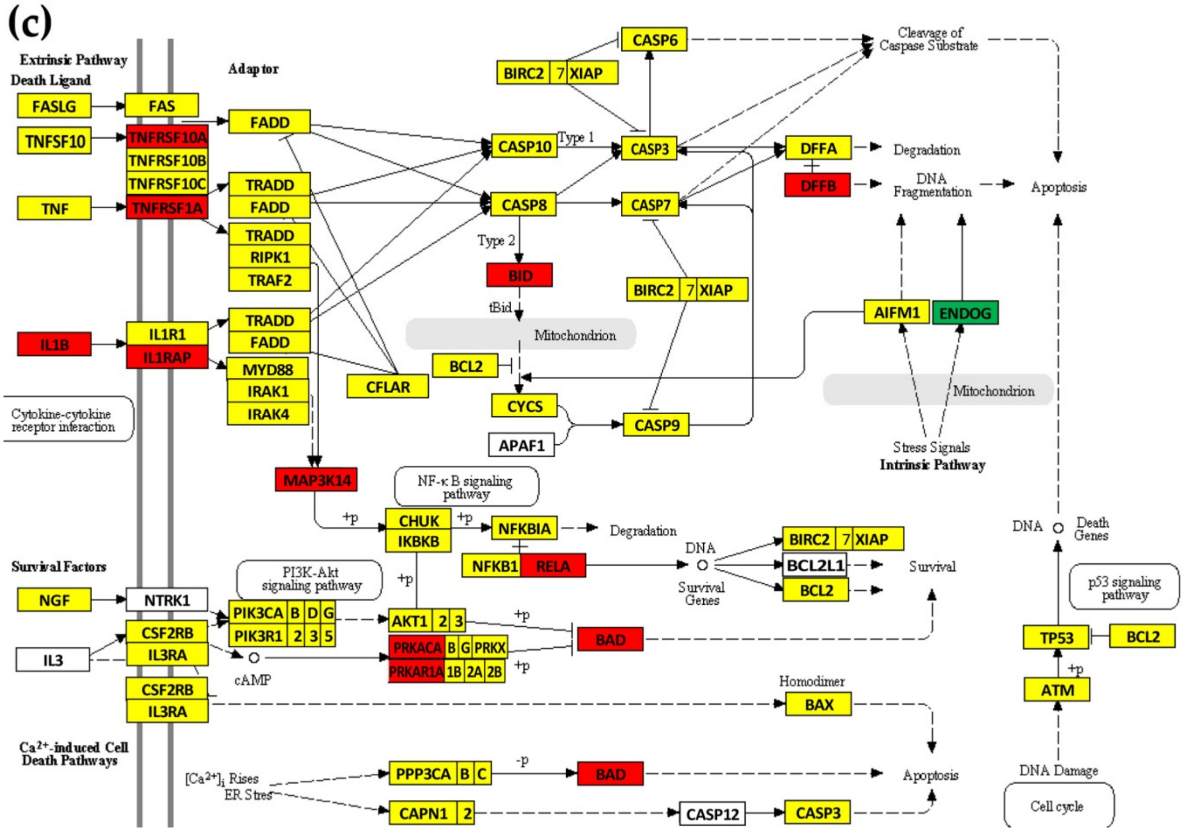

Figure S1. Regulation of the apoptosis pathway in: (**a**) PTA, (**b**) PTB and (**c**) CWM regions with respect to NOR. Regulated genes: *AIFM1* (apoptosis-inducing factor, mitochondrion-associated, 1), *AKT1/3* (v-akt murine thymoma viral oncogene homologs), *BAD* (BCL2-associated agonist of cell death), *BCL2* (B-cell CLL/lymphoma 2), *BID* (BH3 interacting domain death agonist (*BID*), *DFFA*/*B* (DNA fragmentation factors, 40kDa, polypeptide (caspase-activated DNase), *ENDOG* (endonuclease G, *IL1B* (interleukin 1, beta), *IL1RAP IL3RA* (interleukin receptors), *MAP3K14* (mitogen-activated

protein kinase kinase kinase 14), *PPP3CA* (protein phosphatase 3, catalytic subunit, alpha isozyme), *PRKACA* (protein kinase, cAMP-dependent, catalytic, alpha), *PRKAR1A/B* (protein kinases, cAMP-dependent, regulatory, type I), *RELA* (RELA proto-oncogene, NF-kB subunit), *RIPK1* (receptor interacting serine/threonine kinase 1), *TNFRSF1A/10A/10B/10C* (members of the tumor necrosis factor receptor superfamily), *TRAF2* (TNF receptor-associated factor 2).

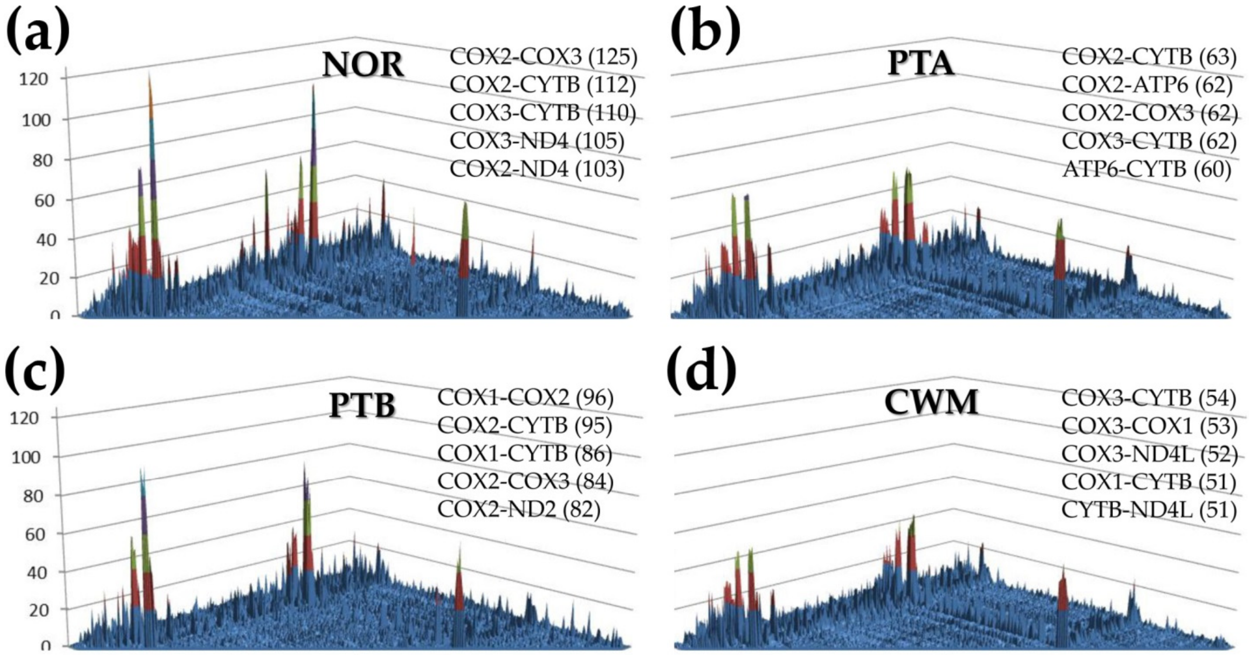

GENE	DESCRIPTION
ATP6	mitochondrially encoded ATP synthase 6
COX1	mitochondrially encoded cytochrome c oxidase I
COX2	mitochondrially encoded cytochrome c oxidase II
COX3	mitochondrially encoded cytochrome c oxidase III
CYTB	mitochondrially encoded cytochrome b
ND2	mitochondrially encoded NADH dehydrogenase 2
ND4	mitochondrially encoded NADH dehydrogenase 4
ND4L	mitochondrially encoded NADH dehydrogenase 4L

Figure S2. The PWR landscapes of mitochondrial genes in the four profiled regions. In each panel, the medallion lists the most relevant five pairs in that region. Genes of the most relevant pairs: *ATP6* (mitochondrially encoded ATP synthase 6), *COX1/2/3* (mitochondrially encoded cytochrome c oxidase I/2/3), *CYTB* (mitochondrially encoded cytochrome b), *ND2/4/4L* (mitochondrially encoded NADH dehydrogenase 2/4/4L).

© 2020 by the authors. Licensee MDPI, Basel, Switzerland. This article is an open access article distributed under the terms and conditions of the Creative Commons Attribution (CC BY) license (http://creativecommons.org/licenses/by/4.0/).
